# Mechanical and Ultrasonic Pretreatments for Efficient Peeling of Metal Foils from Spent Lithium-Ion Batteries

**DOI:** 10.1021/acsomega.4c10547

**Published:** 2025-03-11

**Authors:** Weichen Yang, Zheng Tong, Xiangning Bu, Lisha Dong, Saeed Chehreh Chelgani

**Affiliations:** † Key Laboratory of Coal Processing and Efficient Utilization (Ministry of Education), School of Chemical Engineering and Technology, China University of Mining and Technology, Xuzhou 221116, China; ‡ Western Australian School of Mines: Minerals, Energy, and Chemical Engineering, Curtin University, Kalgoorlie, Western Australia 6430, Australia; § Minerals and Metallurgical Engineering, Swedish School of Mines, Department of Civil, Environmental and Natural Resources Engineering, Luleå University of Technology, Lulea 971 87, Sweden; ∥ Wallenberg Initiative Materials Science for Sustainability, Department of Civil, Environmental and Natural Resources Engineering, Swedish School of Mines, Luleå University of Technology, Lulea 971 87, Sweden

## Abstract

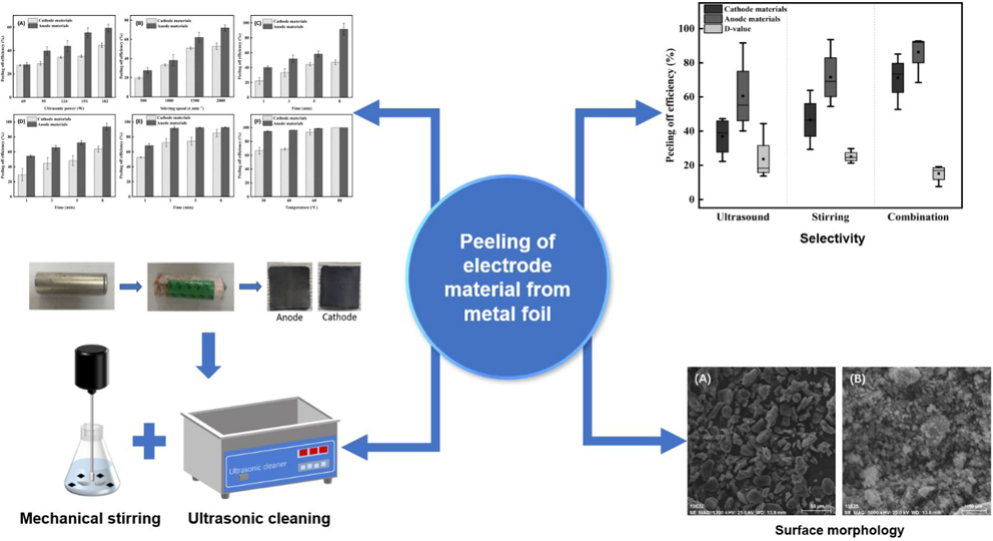

Typical recycling processes of electrode materials of spent lithium-ion batteries are complicated, energy-consuming, have limited separation efficiency, and cause environmental issues. Therefore, examining various environmental approaches, such as physical pretreatments, would be essential to enhance recycling efficiency. As a novel approach in this study, the ultrasonic treatment and mechanical stirring were examined to explore the potential of selective stripping of cathode and anode materials of spent lithium-ion batteries. The effects of various factors on the stripping efficiency and selectivity were assessed (ultrasonic power, mechanical stirring speed, processing time, and temperature). Outcomes indicated that the cavitation generated by ultrasound and mechanical stirring could impact the diffusion process of the aqueous medium. This phenomenon could lead to a high peeling performance of electrode materials, while this effect would be more evident as the intensity of the corresponding parameter was increased. Generally, the overall peeling efficiency for anode materials was higher than for cathode ones in various conditions. Mechanical stirring speed could improve the peeling efficiency of cathode materials. Experimental outcomes demonstrated that the corrosion of metal foils would appear by increasing the intensity of corresponding parameters. Combining ultrasound and mechanical stirring could markedly enhance the peeling efficiency of both cathode and anode materials. In other words, combining these treatments would decrease the peeling selectivity. Various characterizations, such as scanning electron microscope and energy-dispersive X-ray spectroscopy, X-ray diffraction, and X-ray fluorescence, were applied to verify the experimental outcomes.

## 1 Introduction

Since the production of electric vehicles and electronic mobile devices is increasing exponentially, a substantial quantity of used lithium-ion batteries (LIBs) will soon be disposed of in landfills. The service life of LIBs is approximately three years. After repeated charging and discharging, internal electrode materials are gradually inactivated. If they are not recycled and discarded, destruction will be caused to the local environment.^1^ Therefore, LIB recycling has become a necessity since it reduces energy consumption, alleviates the shortage of critical minerals such as Li, Ni, and Co, eliminates pollution from harmful components, and achieves a sustainable development of related industries such as the electric vehicle manufacturing industry.^2^ LIBs consist of a casing and an internal cell. The casing is made of stainless steel or plastic. The internal cell of the battery is a roll-type structure and is mainly composed of the cathode, anode, diaphragm, and electrolyte. Among them, the cathode material is mainly lithium oxide salts, such as lithium cobaltate, lithium iron phosphate, lithium manganate, etc., with aluminum/copper (Al/Cu) foil as the substrate and bonded together with a conductive agent through a binder (such as poly(vinylidene fluoride) (PVDF)). The anode material (such as graphite) is bonded to the copper (Cu) foil by a binder (such as styrene–butadiene rubber (SBR)).^3^ Due to differences in adhesion and water solubility between PVDF and SBR, the anode material can fall off more quickly than the cathode material, providing theoretical guidance for separating and recovering electrode materials of spent LIBs. The composition of spent LIBs is complex and significant in resourcization and contamination. A systematic recycling process should be explored to avoid potential contamination and realize their resuscitation, providing appropriate economic and social benefits and achieving sustainable development of critical metal resources.^4^

In recent years, the recycling process of LIB electrode materials has been gradually improved, including technologies of pretreatment, leaching, chemical purification, repair, and regeneration.^5,6^ The pretreatment step is the most critical part of the recycling flowsheet since its efficiency would directly affect the other downstream processes. Commonly used pretreatment processes include predischarge, mechanical crushing, screening, pyrolysis, alkali dissolution, froth flotation, and other methods to collect electrode materials through beneficiations.^7^ However, these traditional methods are complex in processing, high in energy consumption, and prone to producing toxic and harmful substances, which are no longer suitable for sustainable development.^3^ Therefore, various mechanical pretreatment methods, such as ultrasound as a clean energy method, have been examined to assist in separating electrode materials.Lei, Aldous, Hartley, Thompson, Scott, Hanson, Anderson, Kendrick, Sommerville, Ryder, and Abbott^8^ reported that active material’s separation efficiency from the current LIBs collector can be promoted using high-power ultrasound. Through crushing, ultrasonic cleaning, acid leaching, and precipitation, 97% of Li and 99% of Co were successfully recovered.^9^ Guo, Xia, Mao, and Ding^10^ found that the effective separation of cathode materials from Al foil can be finalized via combined treatments of ultrasound and strong polar solvents (Dimethylacetamides (DMAc and DMF), *N*-methyl-2-pyrrolidone (NMP)). The Al foil can be obtained directly after separation, avoiding subsequent processing and simplifying the process flowsheet. NMP also indicated a high stripping efficiency of cathode material of 99% under a processing temperature of 70 °C, ultrasonic power of 240 W, and processing time of 90 min.^11^ In our recent work, it is demonstrated that combining the ultrasonic wave and mechanical stirring to treat waste graphite anode sheets assisted in recovering Cu foil from anode sheets with high efficiency, and regenerated anode materials also had a high purity.^2^ This might be because the combined ultrasound and mechanical stirring treatments improve the experimental processing efficiency.^12^ However, the effects of ultrasonic waves and mechanical stirring on the peeling process of cathode and anode materials have not been thoroughly addressed. Furthermore, it is notable that differences in peeling efficiencies of active materials between anode and cathode sheets under ultrasonic wave and mechanical stirring have not been investigated.

To fill the knowledge gap, this study aims to systematically illustrate differences in the separation selectivity between the ultrasonic treatment and mechanical stirring on the peeling process of cathode and anode materials. Experiments were carried out under conditions of single ultrasonic treatment, single mechanical stirring, and combined treatments of ultrasound and mechanical stirring to investigate changes in the peeling efficiency of electrode materials. Conductivity and cavitation experiments were used to explore the working mechanism of ultrasonic treatment and/or mechanical stirring. The scanning electron microscopy-energy dispersive spectroscopy (SEM-EDS) images were used to observe the detachment of electrode materials and the loss of metal foil. The X-ray diffraction (XRD) analysis verified the purity and recycling potential of peeled cathode and anode materials. The X-ray fluorescence (XRF) analysis further detected the elemental composition of cathode materials.

## 2 Materials and Methodology

### 2.1 Materials

The spent ternary LIBs (18650 type) were collected from battery recycling stations in Xuzhou, China. Spent LIBs were discharged for 24 h with NaCl solution (4.27 mol/L) until the voltage was lower than 1.5 V, and batteries were then taken out and dried. After being discharged and cathode and anode materials being manually dismantled, they are cut into a square sheet with a width of about one cm for subsequent experiments. Electrode material samples used in the characterizations were wet sieved, dried, and stored in a nitrogen (N_2_) filled reagent bag to minimize/avoid oxidation at room temperature for subsequent use.^2^

### 2.2 Peeling Process

Ultrapure water with a resistivity of 18.2 MΩ·cm was used as the medium in all experiments. One gram of electrode sheets was weighed each time, placed in a 250 mL Erlenmeyer flask, and fixed with clamps. The ultrasonic cleaner and mechanical stirrer’s operational parameters (temperature, power intensity, stirring speed, treatment time/duration) were adjusted according to experimental conditions. After each test, electrode sheets were poured from the Erlenmeyer flask, cleaned, and collected from the filtration paper. Meanwhile, stripped cathode and anode materials were filtered and collected using a vacuum filter. After that, they dried in the oven at 80 °C for 12 h. Dry samples were weighed, and the stripping efficiency of electrode materials was calculated (eq 1)
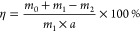
1Where η—the peeling efficiency; *m*_0_—the mass of the filtration paper; *m*_1_—the mass of the sample weighed before experiments; *m*_2_—the mass of the sample weighed after experiments; *a*—the maximum peeling efficiency calculated via the formula for this group of experiments.

The peeling experiment processes were carried out in a mechanical stirrer (JJ-1B, Changzhou Aohua Instrument Co., Ltd.), an ultrasonic cleaner (Skymen JM-07D-40, Skymen Cleaning Equipment Shenzhen Co., Ltd.), and the combined process of a mechanical stirrer and an ultrasonic cleaner.^2^ All experiments were carried out with replicates, and their means and statistical errors of means were reported.

### 2.3 Cavitation Intensity

Interactions of ultrasonic treatment and mechanical stirring will cause cavitation effects and produce hydroxyl radicals with oxidative properties. The “iodine release method” was used to reflect the cavitation effect of ultrasound. Since iodide (*I*^–^) can be oxidized to elemental Iodine under the cavitation, elemental Iodine and iodide ions will form *I*_3_^–^. The determination of *I*_3_^–^ concentration can reflect ultrasonic cavitation strength. In this experiment, an ultraviolet (UV) spectrophotometer was used to detect the absorbance of *I*_3_^–^ at a wavelength of 350 nm. The Potassium Iodide (KI) solution with a concentration of 0.2 mol/L was prepared for the analysis. To be consistent with experimental conditions set for peeling experiments, differences in cavitation strength produced by the ultrasonic treatment, mechanical stirring, and combined treatments were measured and compared, and then differences in the peeling efficiency were verified.^2^

### 2.4 Conductivity Measurement

In addition to the cavitation effect, flow conditions in the flow field can affect the uniformity of energy transfer and then change the peeling efficiency.^2^ The conductivity meter (DDSJ-308F, INESA Scientific Instrument Co., Ltd.) was used to test flow conditions in the flow field under various experimental ultrasonic treatment and mechanical stirring conditions. The detection probe was fixed at the same position. A transparent square glass tank with a side length of 10 cm replaced the Erlenmeyer flask as an experimental container. Before starting each experiment, ultrapure water was added to the ultrasonic tank at the same water level in a transparent glass tank. The conductivity probe and the stirring rotor were fixed at the same position, making the bottom end of the probe close to the liquid surface. The data recording was started simultaneously with the ultrasonic or mechanical stirring experiments. A potassium chloride (KCl) solution with a molar concentration of 2.68 mol/L was prepared, and 10 mL of the solution was measured and injected from the bottom of the container, making the solution diffuse from the bottom to the probe. The automatic counting mode was set, and the conductivity value was recorded every 5 s and counted for 10 min. Then, the recorded data was exported, which reflected the fluctuation of fluid conductivity with time through various experimental conditions of ultrasonic treatment, mechanical stirring, and their combination.

### 2.5 Characterizations

The peeled-off *s*amples were characterized using an X-ray fluorescence spectrometer (XRF, Bruker S8 Tiger, Germany). The peeled-off samples were analyzed using a scanning electron microscope (SEM FEI QUANTA 250, FEI Company) incorporated with an energy-dispersive X-ray spectrometer (EDS). The SEM images were obtained at 20 kV accelerating voltage. The X-ray diffraction method analyzed the peeled-off sample (D8 Advance, Bruker Company, Germany). XRD patterns were obtained with a D/MAX-2500 pc powder diffractometer using Cu–Kα (λ= 1.54 Å) radiation generated at 40 kV and 40 mA at China University of Mining and Technology, Xuzhou.

## 3 Results and Discussions

### 3.1 Peeling Efficiency

#### 3.1.1 Single Ultrasonic Treatment

**Figure 1:**
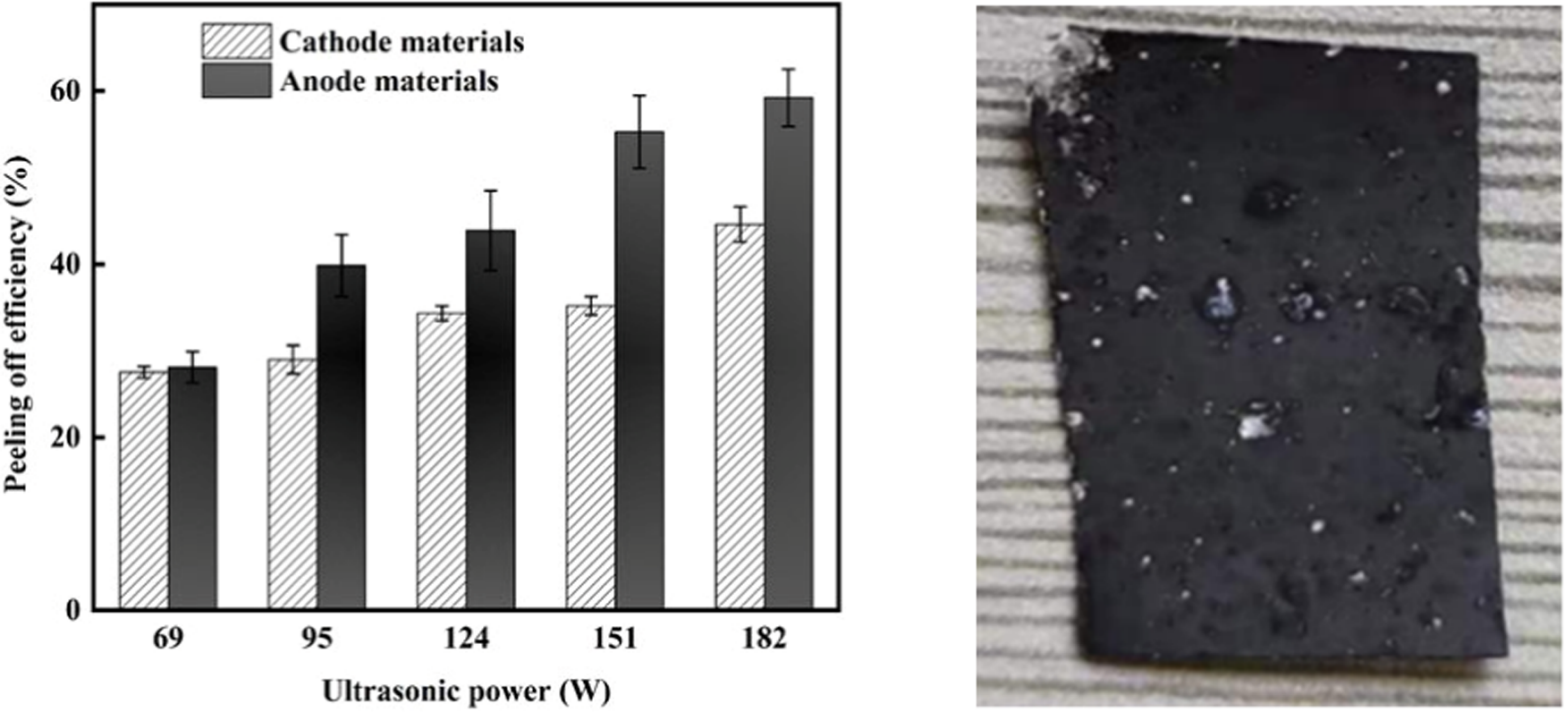
Effects of various ultrasonic powers on peeling efficiency and corrosion on Al foil under the ultrasonic treatment with an ultrasonic power of 151 W.

Ultrasonic power was set at different gears based on our device limitation range (69, 95, 124, 151, and 182 W), at a constant temperature (25 °C) and ultrasonic treatment duration (5 min). It can be seen from Figure 1 that the stripping efficiency of electrode materials from Cu and Al foils shows an upward trend with the increase of ultrasonic power and treatment duration, and the stripping efficiency of anode materials from Cu foil is generally higher than that of cathode materials from the Al foil, which reflects differences in the binder adhesion for cathode and anode materials. As the ultrasonic power increases, the concentration of cavitation bubbles increases, and the energy generated by the collapse of cavitation bubbles increases, strengthening the cavitation effect and increasing the stripping efficiency of electrode materials.^13^ However, when ultrasonic power is too high, many invalid bubbles are produced, forming a sound barrier and attenuating the scattering of ultrasonic waves. This may reduce the stripping efficiency of electrode materials and cause corrosion of the metal foil. Excessively high ultrasonic power should be avoided in experiments.^11^ When ultrasonic power was 151 W, partial corrosion started to appear on Al foil, while there were no signs of corrosion on Cu foil (Figure 1), which indicated Al foil was easier to corrode than Cu foil through the same experimental conditions.

**Figure 2:**
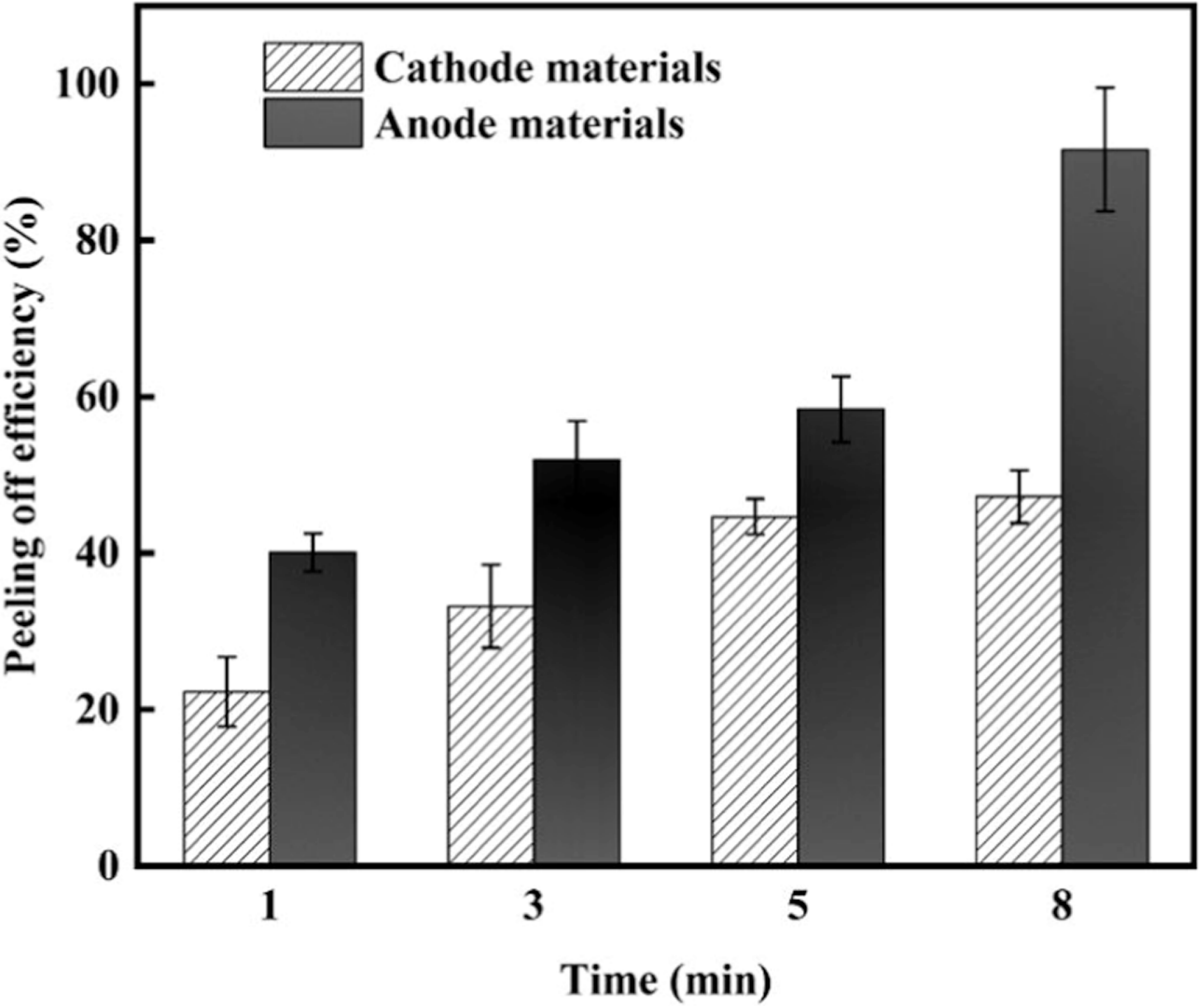
Effects of various ultrasonic treatment durations on the peeling efficiency.

Fixing ultrasonic power at 182 W, experiments were carried out with different ultrasonic treatment durations (1, 3, 5, and 8 min). Results (Figure 2) illustrated that increasing the ultrasonic treatment duration increased the stripping efficiency of anode material. This increase was significant at 8 min compared to 5 min. However, the increase in stripping efficiency of cathode materials from Al foil was insignificant through different durations. These outcomes demonstrated that the PVDF binder for cathode materials was debonded to a lower degree, which hindered the peeling of cathode materials from Al foil. Therefore, different optimal experimental conditions would be required to explore. Differences in the peeling efficiency of electrode materials from Cu and Al foils tended to become larger with the increase of ultrasonic power and treatment time, indicating that the ultrasound had different effects on the selectivity of peeling processes for cathode and anode materials. It is difficult to dissolve the organic binder, mainly PVDF, utilized in the cathode sheet in water. As a comparison, the organic binder used for the anode sheet is water-soluble, such as styrene–butadiene rubber (SBR) and sodium carboxymethyl cellulose (CMC).^14,15^ Thus, the differences in the peeling efficiencies between anode and cathode electrode plates can be attributed to the different organic binders.

#### 3.1.2 Single Mechanical Stirring Treatment

**Figure 3:**
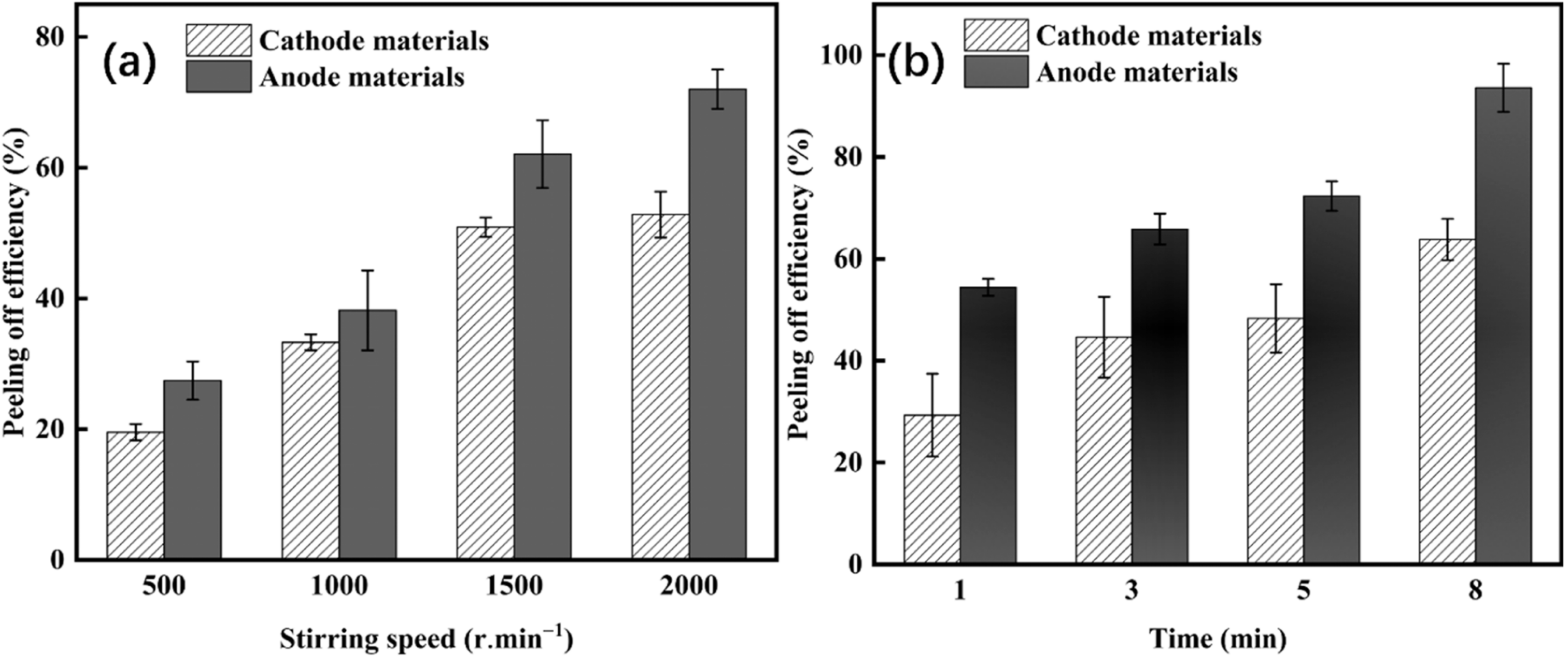
Effects of mechanical stirring speed and mixing time on the peeling efficiency, the effect of (a) mechanical stirring speed; (b) mixing time.

The stirring speed was set at different gears based on our device limitation range (500, 1000, 1500, and 2000 rpm) at a constant operation temperature (25 °C) and treatment duration (5 min). Experimental results (Figure 3a) indicated increased peeling efficiency by increased stirring speed. This increase was higher for anode materials compared to cathode materials. In addition, with the increase in rotation speed and time, stripping efficiency showed an upward trend. This enhancement of the higher rotation speed can be attributed to the mass transfer enhancement caused by the stronger flow convection.^16^

**Figure 4:**
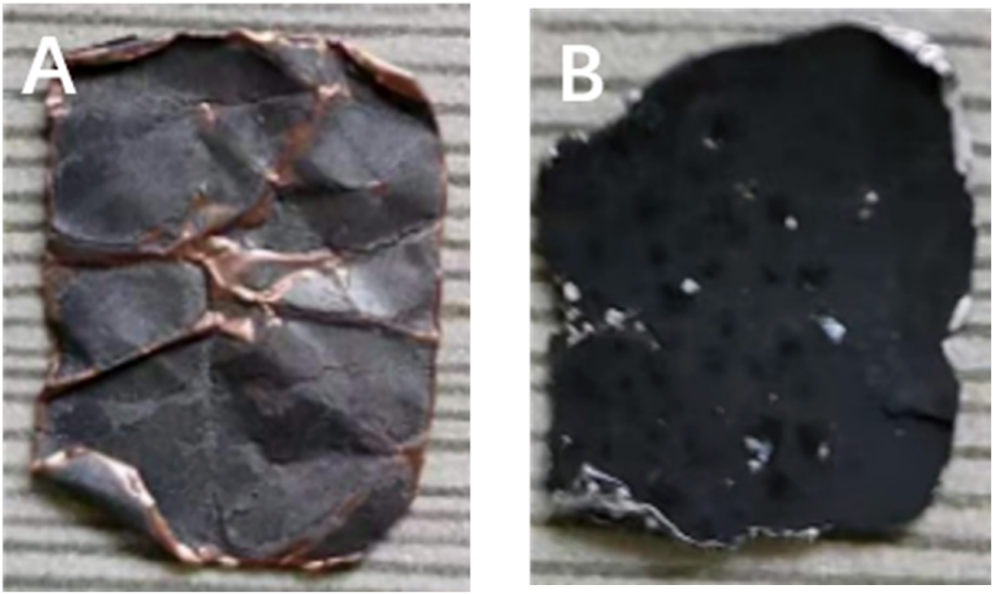
Corrosion appeared on Cu and Al foil at a stirring speed of 2,000 rpm, (A) Cu foil; (B) Al foil.

Various mechanical stirring treatment durations (1, 3, 5, and 8 min) with a constant speed (2000 rpm) showed (Figure 3b) that by increasing the treatment time, the peeling efficiency would be enhanced for both anode and cathode materials. However, under the same treatment time (8 min), the differences in the stripping efficiency of cathode and anode materials with mechanical stirring were lower than those with ultrasonic treatment. When the stirring speed increased to 1000 rpm, variations in the appearance of Cu and Al foil could be observed (Figure 4). Excessive rotational speed can cause the electrode sheets to become curled. In addition, the edges and corners of the electrode sheet material will be removed by the stirring blade.

#### 3.1.3 Combined Treatment—Ultrasound and Mechanical Stirring

**Figure 5:**
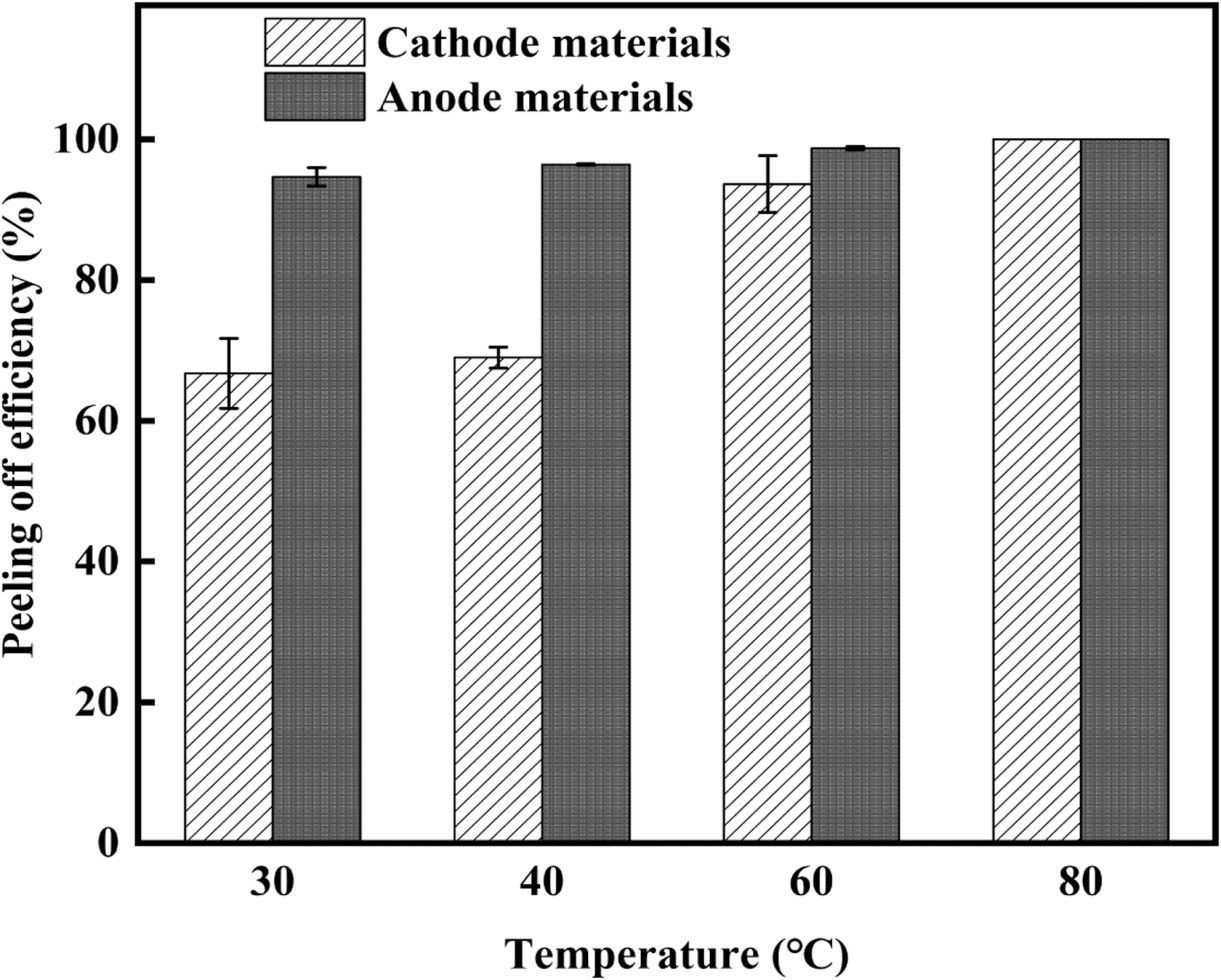
Effects of operation temperature on the stripping efficiency. The error bar stands for two independent runs.

Experiments were continued by combining ultrasound and mechanical stirring treatments at different temperatures (30, 40, 60, and 80 °C) while the power (182 W) and stirring speed (2000 rpm) were constant for a 5 min process. Assessments of experimental outcomes indicated (Figure 5) that the peeling efficiency of electrode materials from Al and Cu foils was markedly higher in the combining treatment methods compared with the single individual ultrasonic or mechanical stirring treatment. The peeling efficiency was maximum at 80 °C, indicating cooperative effects of ultrasonic and mechanical stirring treatments improved the separation process. This could be due to the combined impact of strengthening the cavitation effect and/or convective movement of fluids, thereby promoting the generation and transfer of energy during experiments.^16^ As the temperature increased (Figure 5), the stripping efficiencies of electrode materials from the Al and Cu foils gradually approached each other, and both finally reached the maximum, showing that high temperature (>60 °C among all tested temperatures) reduced the selectivity in stripping efficiencies of electrode materials from Al and Cu foils.

**Figure 6:**
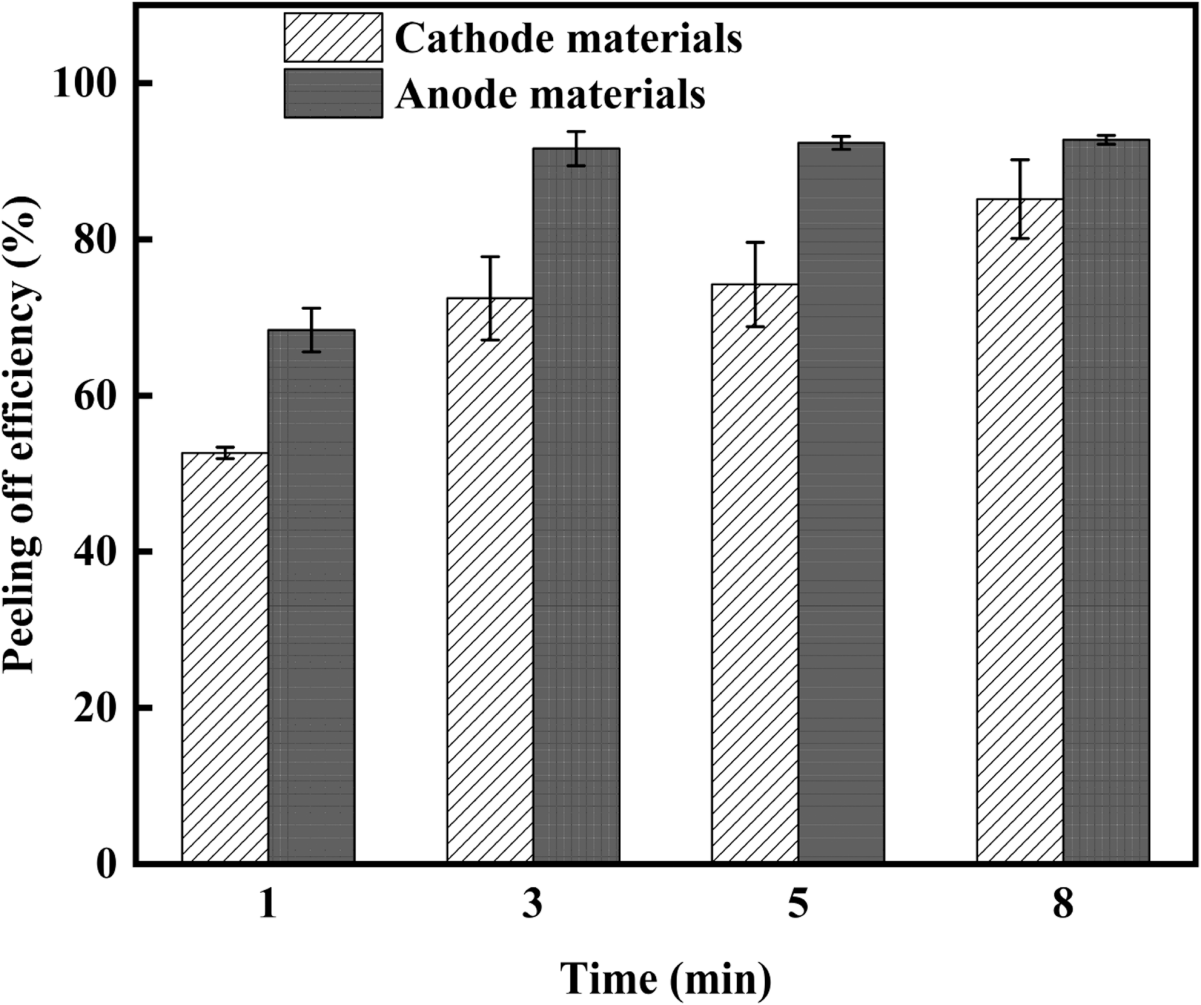
Effects of treatment time on the peeling efficiency. The error bar stands for two independent runs.

Various experiments with different treatment durations (1, 3, 5, and 8 min) were conducted to explore the effect of processing time on the combined treatments. At the same time, the other parameters were constant (temperature 25 °C, power 182 W, and stirring speed 2000 rpm). Experimental outcomes (Figure 6) highlighted that increasing treatment time increased the peeling efficiency for both anode and cathode materials, and differences between their peeling efficiencies were significantly decreased (lower selectivity). By optimizing joint processing parameters—such as ultrasonic power, stirring speed, processing time, and temperature—this method can be effectively applied to the stripping and processing of various types of battery materials at an industrial scale. Such optimization enhances the resource recovery rate of spent LIBs, resulting in more significant economic benefits and reduced environmental pollution.

While combined treatments demonstrated superior overall peeling efficiencies, there are specific scenarios where single treatments may be more practical. For example, when processing batches predominantly composed of anode sheets, a single ultrasonic treatment could suffice due to the water-soluble nature of SBR binders, minimizing energy input and operational costs. Similarly, single mechanical stirring may be appropriate for applications prioritizing cathode peeling efficiency, given its stronger performance in detaching cathode materials from aluminum foils. These targeted approaches reduce energy and material waste and decrease the risk of overpeeling, which could lead to increased contamination from corroded metal foils. Optimizing operational setups tailored to the composition of the spent battery materials could provide a balanced trade-off between efficiency, cost, and environmental impact.

### 3.2 Peeling Mechanism Investigation via Various Characterizations

#### 3.2.1 Cavitation Experiment

**Figure 7:**
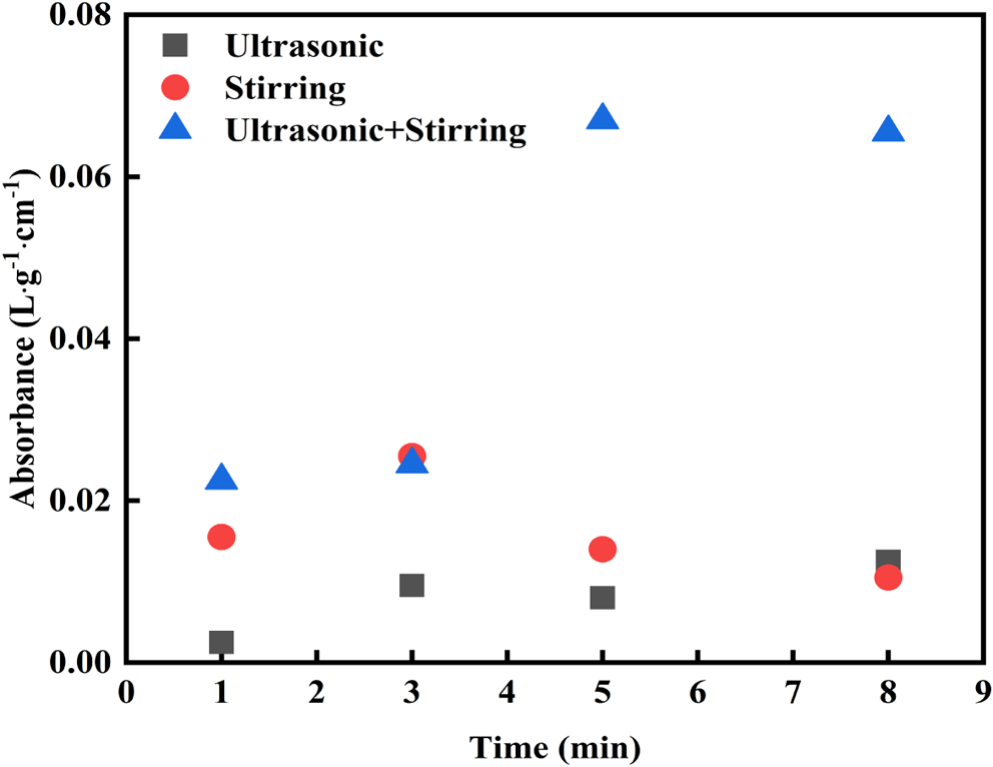
Results of cavitation strength tests with ultrasonic treatment, mechanical stirring, and combined treatments.

Cavitation experiments were carried out under the experimental condition with a fixed ultrasonic power of 182 W, mechanical stirring speed of 2000 rpm, and temperature of 25 °C. The KI solution with a molar concentration of 0.2 mol/L was added to the Erlenmeyer flask, and the amount was the same as that used as the medium for peeling experiments (200 mL). During the cavitation process, Iodide ions are oxidized by hydroxyl radicals to elemental Iodine, which then reacts with Iodide ions to form Iodide triions (*I*_3_^–^). The chemical reaction involved is listed in eq 2
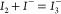
2After being treated under each experimental condition, the absorbance of *I*_3_^–^ in the solution at a wavelength of 350 nm was immediately detected. As the experimental results illustrated (Figure 7), the absorbance of *I*_3_^–^ under the combined treatment was greater than that under the single ultrasound or mechanical stirring treatment, especially with treatment durations of 5 and 8 min, when the difference was significantly obvious. The absorbance of *I*_3_^–^ in the solution through ultrasound treatment was slightly higher than that under the single treatment of mechanical stirring with the same treatment duration of 8 min and was lower than that with the single treatment of mechanical stirring under experimental conditions of other tested treatment durations. It could be concluded that the cavitation intensity produced by the combined ultrasound and mechanical stirring treatments was higher than each treatment, and differences were more evident after being treated for 5 min and/or a longer period. The cavitation intensity under ultrasonic treatment was lower than the mechanical stirring one when the treatment duration was less than 8 min and slightly higher than the latter after being treated for more than 8 min.

#### 3.2.2 Conductivity Measurement

**Figure 8:**
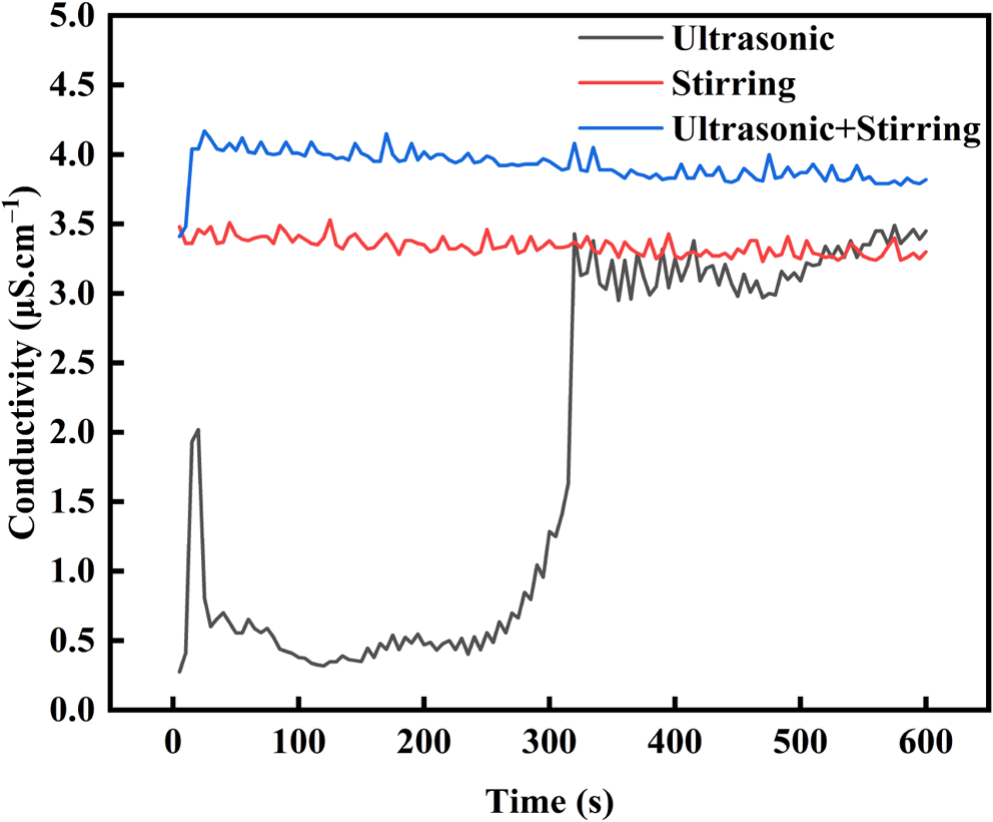
Results of ion conductivity tests under single ultrasonic treatment or mechanical stirring and combined treatments.

The conductivity measurements of solutions were carried out under single ultrasonic treatment or mechanical stirring and combined treatments, and the relationship between the conductivity of the solution and time. Measurements (Figure 8) indicated that the conductivity of the solution under combined treatments of ultrasound and mechanical stirring was greater than that under the individual treatment. In addition, the conductivity of the solution reached a high value of 4.0 μS·cm^–1^ with combined treatments or single treatment of mechanical stirring when it started to time, and there was no obvious evidence indicating the diffusion process of K^+^ and Cl^–^ ions from the bottom to top. However, an apparent upward diffusion process at 0 s was observed through ultrasonic treatment, which started to fluctuate up and down after being treated for 300 s and then tended to be stable. It highlighted that the effect of ultrasound on the diffusion and turbulence of fluids was relatively low (not as high as the mechanical stirring treatment).

Compared to single individual treatment, combined treatments with a higher cavitation intensity could produce more free radicals, which was effective for the degradation of organic binder. Meanwhile, the combined treatment could induce stronger liquid convection into the bath to ensure a fast and sufficient supply of free radicals into the metal foil surface. These phenomena confirmed the higher peeling efficiency of combined treatments than the individual ones and showed their strong synergic effect in the peeling electrode materials from metal foils.

**Figure 9:**
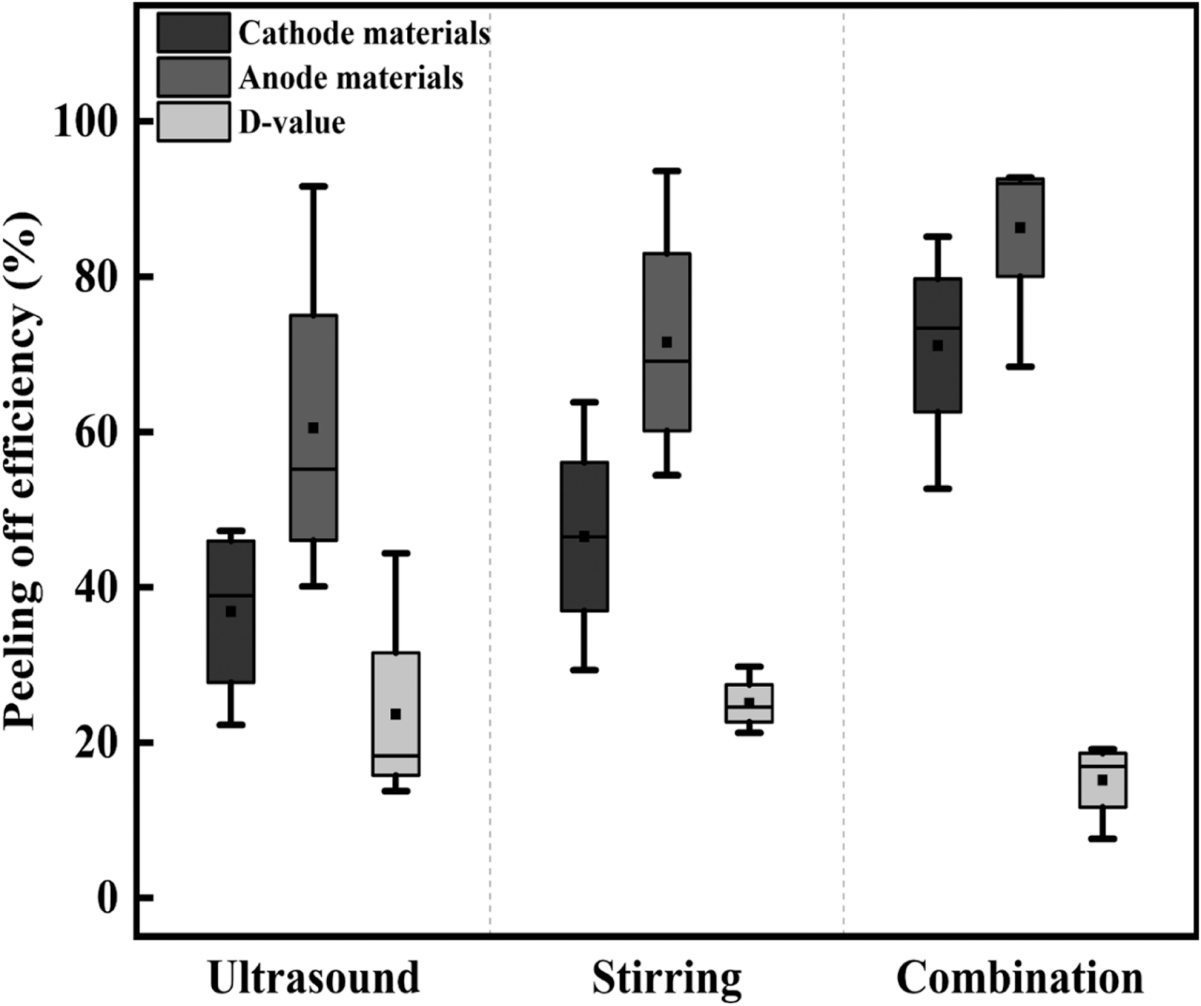
A general comparison between the peeling selectivity of various treatment conditions.

Box plots of treatment outcomes in various conditions for the single ultrasound, the single mechanical stirring, and the combined treatments are given in Figure 9. It was highlighted that ultrasonic treatment generally could result in higher peeling efficiency than stirring speed for anode material, while for cathode materials stirring speed provided a higher efficiency. Meanwhile, it is indicated that the selectivity generated with mechanical stirring treatment is greater than that of ultrasonic treatment. These assessments also illustrated that the differences in the stripping efficiencies of electrode materials from Al and Cu foils were lower than those under individual treatment of mechanical stirring. In other words, the peeling selectivity worsened when combined treatment methods. The poor separation selectivity using the combined treatment was related to the significant enhancement in the peeling efficiency of cathode materials from Al foils. As observed from Figures 7 and 8, this enhancement for the combined ultrasound and mechanical stirring can be attributed to the promotion of the generation of free radicals and the improvement in liquid convection.

#### 3.2.3 Surface Morphology and Elemental Analysis

**Figure 10:**
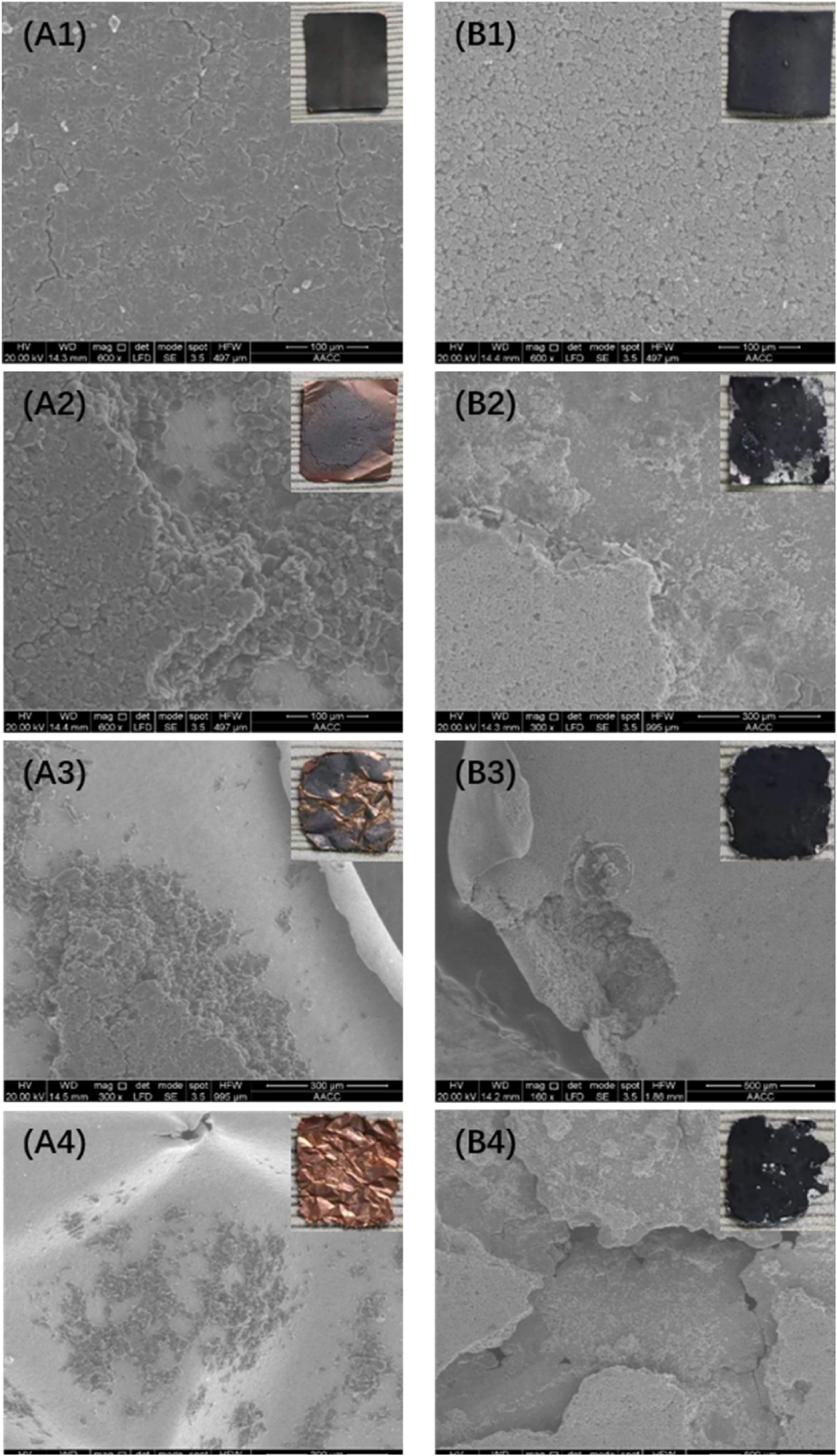
SEM images and photos of cathode and anode materials: (A) Anode; (B) Cathode; (1) Before the experiment; (2) Ultrasonic treatment; (3) Mechanical stirring treatment; (4) Combined treatments.

To further verify the peeling effects on electrode materials and the corrosion to metal foils, the SEM analysis was carried out on electrode sheets before and after experiments (Figure 10). With the ultrasonic treatment, cathode and anode materials were peeled off consistently, and the apparent peeling area (Figure 10A2,B2); with the treatment of mechanical stirring, the edge of the Cu foil is bent (Figure 10A3), and the Al foil (Figure 10B3) was partially damaged, indicating that mechanical force would cause different degrees of damage to metal foils of cathode and anode materials. These issues might be related to the texture of the experiment material. The texture of Cu foil is softer than the Al foil; thus, the degree of damage would be less compared with that of the latter. For the combined treatments, more wrinkles appeared in the Cu foil (Figure 10A4); it was partially broken (more shedding from the anode material). The damage to the Al foil was also aggravated (Figure 10B4), and the shedding rate of cathode materials was also increased. This process was consistent with the photos, verifying the peeling experimental results. XRF analysis for cathode materials (Table 1) demonstrated that the composite elements for the cathode material were mainly Ni, Co, and Mn, which was highly consistent with XRD results. 11.43 wt % Al_2_O_3_ was verified from the XRF analysis, which implied that the Al foil had indeed suffered from corrosion, indicating that the loss entered cathode materials. The loss of Al foils in cathode materials can complicate the subsequent recycling process.^15^ Therefore, joint processing can exacerbate the breakage and damage of copper and aluminum foils. If not managed effectively, contaminants from the damaged metal foils may lead to environmental pollution and hinder subsequent recycling efforts.

**Table 1:** XRF Analysis for Cathode Materials

compound	weight (wt %)	compound	weight (wt %)	compound	weight (wt %)
Al_2_O_3_	11.43	SiO_2_	0.09	Na_2_O	0.10
CaO	3.26	SrO	0.01	NiO	34.98
Cl	0.02	ZnO	0.06	P_2_O_5_	0.83
Co_3_O_4_	17.77	K_2_O	0.02		
Fe_2_O_3_	0.06	MnO	31.35		

**Figure 11:**
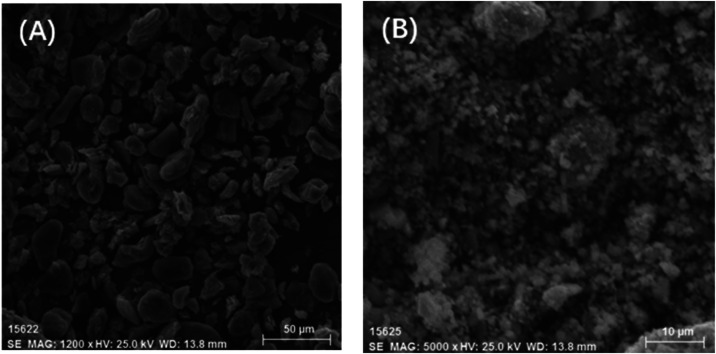
SEM images of stripped cathode and anode materials, (A) anode; (B) cathode.

The stripped cathode and anode materials were collected and sieved through a sieve with an aperture size of 74 μm, and the downsizes were sent for the SEM-EDS analysis. SEM images illustrated (Figure 11) that the dispersion of anode materials was homogeneous, and no agglomeration occurred into large particles. This fact indicated that the SBR binder for the anode materials was damaged to a high degree and dissolved in the water medium. However, the cathode material was agglomerated into many large particles, indicating that after the combined treatment methods, the binder PVDF for the cathode materials still existed in a large quantity, hindering the peeling and dispersion of cathode materials. The corrosion of metal foils might be caused by the ultrasonic treatment with a high powder intensity or mechanical stirring at a high stirring speed,^17^ as shown in Figures 1 and 4.

**Figure 12:**
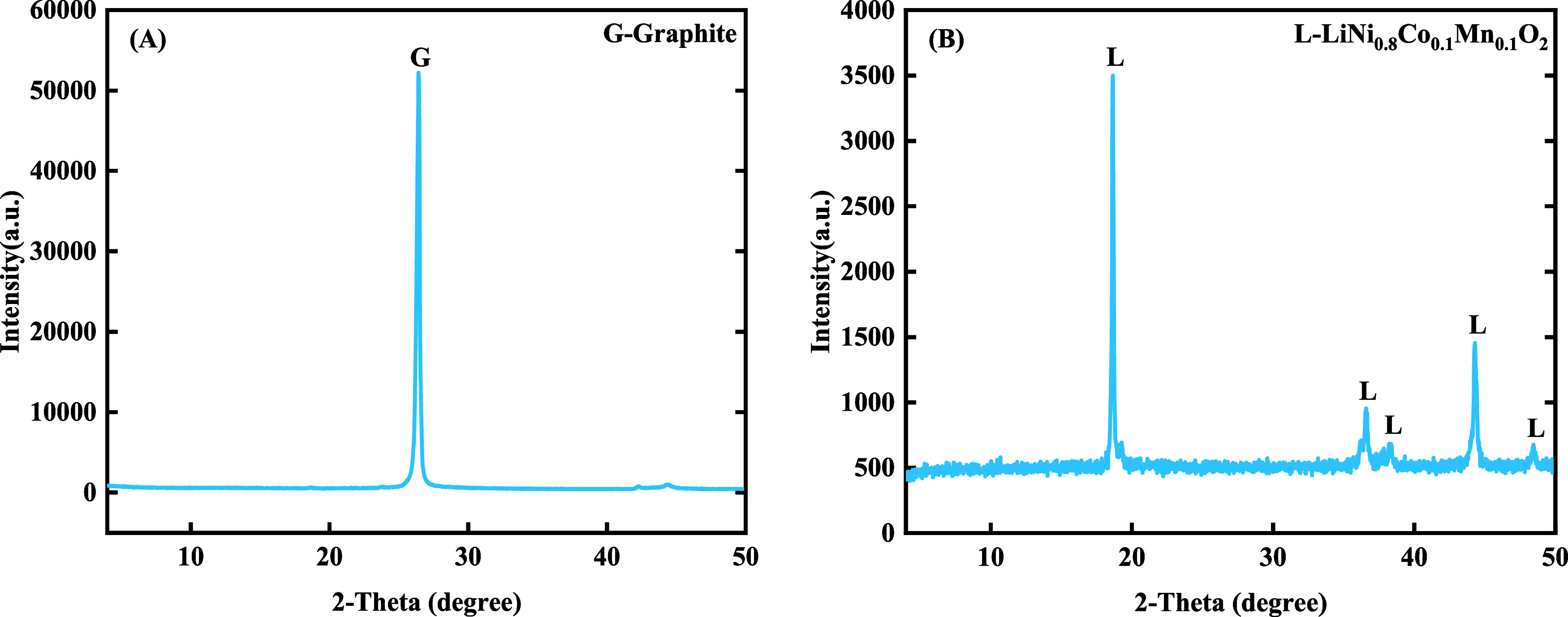
XRD patterns of cathode and anode materials after the stripping process, (A) anode; (B) cathode.

**Figure 13:**
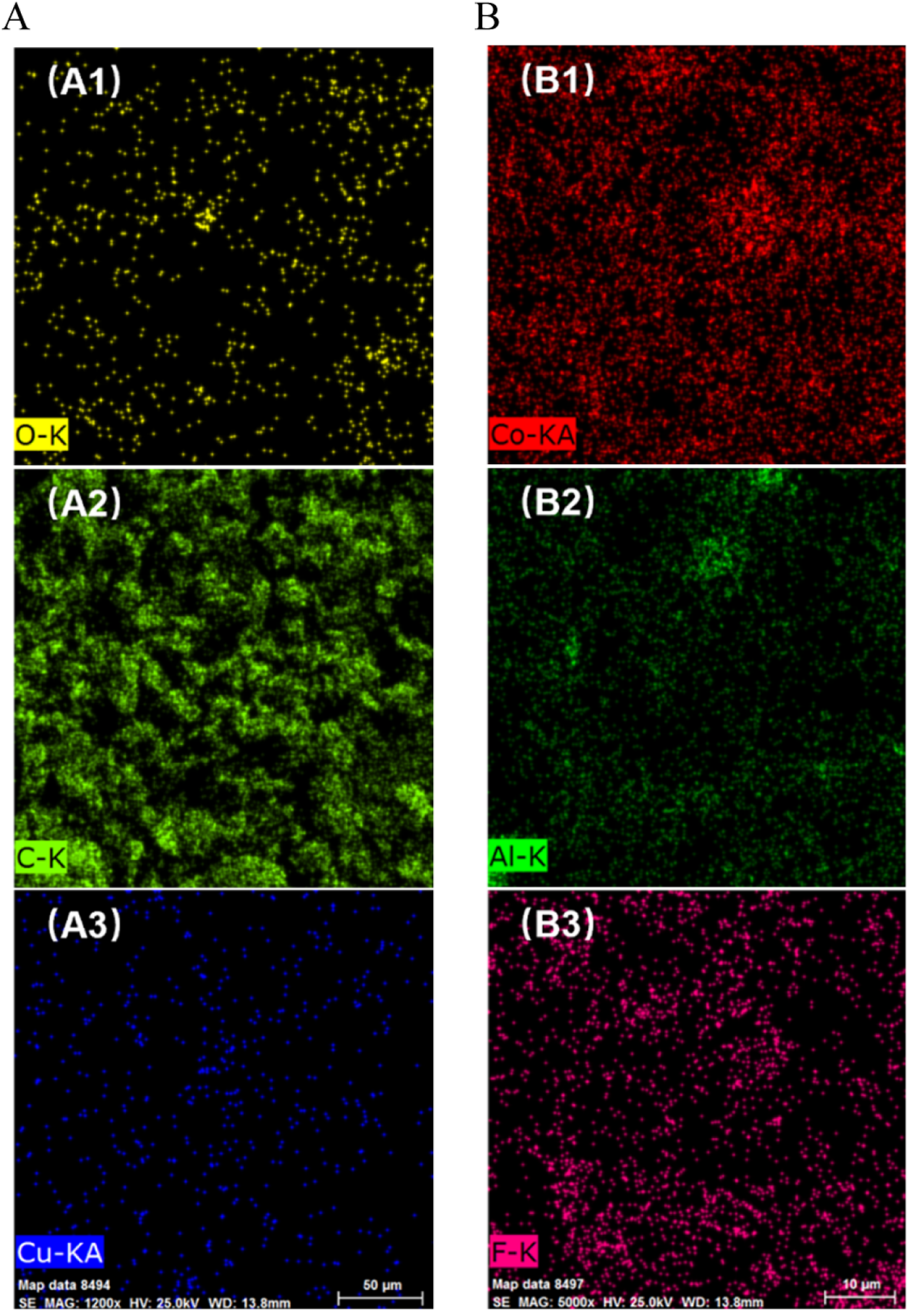
EDS images of (A) anode and (B) cathode materials. A1, A2, and A3 are for the anode materials; B1, B2, and B3 are for the cathode materials.

Theoretically, anode materials should be only graphite. Through the XRD analyses (Figure 12), a small amount of Cu element was detected, indicating that the anode material was slightly oxidized during the period from the dismantling and stripping to the final detection process. A small portion of the fine slag particles of Cu foil produced due to the damage caused by the ultrasonic treatment and/or mechanical stirring produced were mixed into the cathode material. Analyses showed the diffraction peak of the anode material at around 26.5° was consistent with the standard peak positions of graphite with a Powder Diffraction File (PDF) number of 12–0212 (provided by the International Centre for Diffraction Data (ICDD)), indicating the exfoliated graphite had a high purity. However, some miscellaneous peaks demonstrated a slight contamination through the experimental process. Diffraction peaks of LiNi_0.8_Co_0.1_Mn_0.1_O_2_ were mainly detected in XRD analysis of cathode materials (Figure 12(B)), which followed standard peaks with PDF cards of 87–1562 and 88–0657 provided by ICDD. The element ratio of Ni–Co–Mn (NCM) was reasonable (around 8:1:1) during the battery’s multiple charging and discharging processes and the peeling and recycling process. EDS analyses (Figure 13) also illustrated that the Cu (Figure 13A3) and the Al element (Figure 13B2) were less than in other conditions, which further verified the slight pollution of the cathode and anode materials. Thus, a small amount of metal foil polluted both cathode and anode materials, indicating that the stripping and collection process strategy needs to be better optimized.

**Figure 14:**
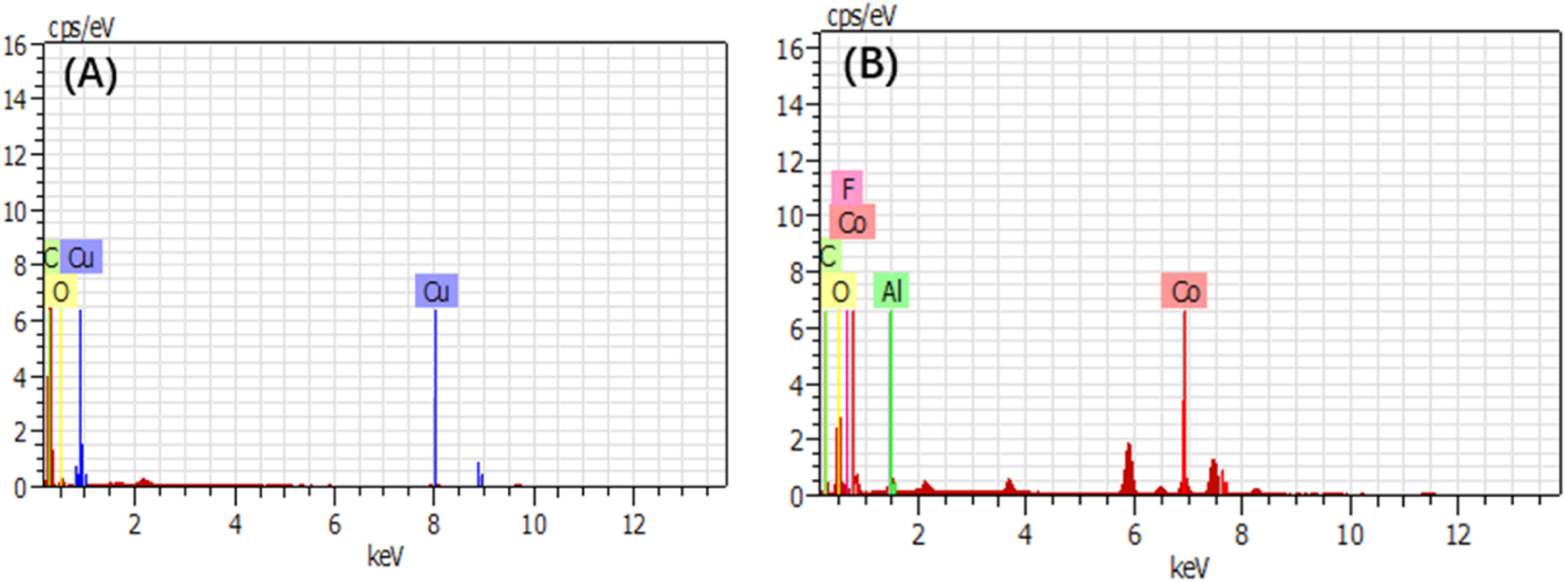
EDS element analysis of cathode and anode materials, (A) anode, (B) cathode.

EDS analyses (Figure 14 and Table 2) also showed that the stripped cathode material contained a small amount of C element, which might be caused by the cross-contamination of anode materials or during the sieving process. The mixture of 3.35 wt % Al element and 8.41 wt % F element (Table 2) verified the conjecture for the existence of agglomerations in the cathode material in SEM images. The destruction of the Al foil caused the appearance of the Al element, and the appearance of the F element was a sign of the existence of a large amount of PVDF. It indicated that the degree of damage to PVDF was small with ultrapure water as the medium, and cathode materials could not be effectively peeled off.^18^ Thus, it is reasonable to speculate that the organic binder for a negative plate was water-soluble because the water-based binder couple CMC/SBR was already commonly used in anodes.^19,20^ PVDF used in cathodes has multiple excellent properties, such as elasticity, low weight, low thermal conductivity, high chemical corrosion resistance, and heat resistance.^21^ Compared to PVDF in cathodes, the water-soluble binder in anodes is more easily distorted, which leads to the superior peeling performance of anode materials from Cu foil (Figure 6).

**Table 2:** Elemental Analysis of Cathode and Anode Materials

type	cathode					anode		
element	O	C	Co	Al	F	O	C	Cu
weight (wt %)	55.92	22.87	9.45	3.35	8.41	11.16	87.78	1.06
atomic (%)	57.05	31.08	2.62	2.03	7.22	8.70	91.10	0.21

## 4 Conclusions and Outlook

In this work, investigations were carried out on the effects of different processing times, ultrasonic power, stirring speed, and processing temperature on the peeling efficiency and selectivity of electrode materials of spent LIBs. Based on various assessments considered in this article, the main conclusions are listed as follows:

- The stripping efficiency increases as the intensity/amplitude of each parameter increases. In general, the peeling efficiency for anode materials of spent LIBs is higher than that of cathode materials, and the separation selectivity for anode materials is better.
- With different ultrasonic power and mechanical stirring speeds, differences in stripping efficiency of cathode and anode materials with mechanical stirring were more significant than with the ultrasonic treatment; with varying times of processing, the selectivity obtained with combined treatments is smaller than that of a single treatment of either ultrasonic or mechanical stirring; and stirring conditions; the temperature also plays an essential role on the stripping selectivity.
- Cavitation effects caused by combined ultrasound and mechanical stirring treatments are higher than those caused by each ultrasonic or mechanical stirring treatment, and the difference is more evident after being treated for more than 5 min.
- Regarding fluidity, conductivity experiments have verified that the ultrasonic treatment has less impact on the flow diffusion and disturbance than mechanical stirring, and the flow liquidity with the ultrasonic treatment is not as evident as with the mechanical stirring.
- Metal foil impurities appearing in peeled products are caused by the corrosion of metal foils treated by ultrasound and/or mechanical stirring with a high stirring speed.
- Differences in stripping efficiency of cathode and anode materials include the water solubility of SBR for anode materials being higher than the PVDF binder for cathode materials in the pure water medium. The medium needs to be changed later to destroy PVDF and improve the stripping efficiency of cathode materials.

Generally, potential postpeeling separation techniques could be density-based separation using hydrocyclones or air classification, which could be implemented to capitalize on the density differences between the lighter anode and heavier cathode materials. Selective froth flotation could also be an efficient anode and cathode separation method. Moreover, selective leaching techniques could target residual impurities in the cathode and anode fractions, improving the overall purity of each material. For example, chemical reagents optimized to dissolve residual PVDF from cathode materials could be integrated into the process without affecting anode materials. These postpeeling separation steps can significantly enhance recovery efficiency and minimize cross-contamination between electrode materials, aligning with sustainable recycling practices.

## 5 Future Studies

This study primarily focused on the water-based separation process for cathode and anode materials in lithium-ion battery recycling, addressing the effects of ultrasound and stirring on binder degradation and material selectivity. While the results provide valuable insights into the mechanism and efficiency of material separation, several aspects warrant further exploration to enhance the understanding and scalability of the proposed method. (1) Quantitative and Visual Evidence of Cavitation Effects: Although the cavitation effects induced by ultrasound were characterized using *I*_3_^–^absorbance, more detailed investigations are necessary to validate the underlying mechanisms comprehensively. Future studies could incorporate real-time imaging or simulations of bubble dynamics to visualize and quantify the interplay between cavitation, convective flows, and binder degradation. This could provide a more precise understanding of how these factors contribute to the peeling efficiency of electrode materials. (2) Binder Degradation Mechanisms: This work highlighted the differences in peeling efficiency between the hydrophobic PVDF binder of cathode materials and the more easily degradable SBR binder of anode materials. However, further studies employing advanced analytical techniques, such as X-ray photoelectron spectroscopy (XPS) or infrared spectroscopy (IR), are needed to identify the functional groups involved in binder degradation and systematically compare their degradation rates. These insights would be instrumental in optimizing process parameters for different binder types. (3) Corrosion of Metal Foils: Copper and aluminum foils were corroded during the process, potentially affecting the purity of recovered materials and the scalability of the method. Future research should quantify the extent of corrosion under various treatment conditions and evaluate strategies to mitigate these effects. Possible approaches include optimizing solution pH, incorporating corrosion inhibitors, or designing systems that minimize oxidative conditions during treatment.

To further improve the process’s scalability and economic feasibility, ongoing work can focus on refining the balance between separation efficiency and material losses. Exploring systems that integrate oxidants or other additives could provide valuable insights into achieving a more selective and less corrosive separation process. Beyond the scope of this study, future investigations can also expand to alternative aqueous and nonaqueous systems to evaluate their efficacy in binder degradation and material recovery. Comparative studies across various electrolytes and solvents would be beneficial for identifying the most efficient and environmentally sustainable approaches. Translating these findings to industrial-scale recycling processes will require comprehensive pilot studies to evaluate the economic and operational feasibility. Addressing issues such as energy consumption, process throughput, and waste management will be critical in advancing the applicability of this method. Addressing these aspects in future research can significantly enhance our understanding of binder degradation mechanisms, material recovery efficiency, and system optimization, ultimately contributing to developing a more effective and sustainable recycling process for lithium-ion batteries.
